# Supplementation of 2-Ap, Zn and La Improves 2-Acetyl-1-Pyrroline Concentrations in Detached Aromatic Rice Panicles *In Vitro*

**DOI:** 10.1371/journal.pone.0149523

**Published:** 2016-02-24

**Authors:** Zhaowen Mo, Jinxia Huang, Di Xiao, Umair Ashraf, Meiyang Duan, Shenggang Pan, Hua Tian, Lizhong Xiao, Keyou Zhong, Xiangru Tang

**Affiliations:** 1 Department of Crop Science and Technology, College of Agriculture, South China Agricultural University, Guangzhou, PR China; 2 Scientific Observing and Experimental Station of Crop Cultivation in South China, Ministry of Agriculture, PR China, Guangzhou, PR China; 3 Wuzhou Municipal Bureau of Agriculture, Wuzhou, Guangxi, China; 4 School of Life Science, Jiaying University, Meizhou, Guangdong, China; Ghent University, BELGIUM

## Abstract

Aromatic rice is highly prized by consumers worldwide due to its special aromatic character. 2-acetyl-1-pyrroline (2-AP) is considered to be the single most important volatile compound responsible for aroma in aromatic rice. The present study demonstrated the effects of 2-AP, zinc (Zn) and lanthanum (La) on the 2-AP concentration of detached aromatic rice panicles *in vitro*. Detached panicles from three well-known aromatic cultivars, Guixiangzhan, Pin14, and Pin 15, were cultured separately in basic culture medium supplemented with 2-AP, Zn and La, and 2-AP concentrations were assessed at 7 and 14 days after culture (DAC). The results show that supplementation of 2-AP, Zn and La in the basic culture medium significantly increases the accumulation of proline. 2-AP concentration and the activity of proline dehydrogenase (ProDH) were also increased in rice grains. Zn concentrations were also found to be higher when Zn was added to the basic culture medium, and La concentrations in grains were too low to be measured. Additionally, grain 2-AP concentrations were significantly and positively correlated with proline concentrations, ProDH activities in grains and 2-AP in culture medium. In summary, higher grain 2-AP concentrations might be due to Zn- and La-induced increases in proline concentrations and ProDH activities, as well as the direct uptake and transportation of 2-AP from the culture medium. Furthermore, application of both Zn and La might be helpful for improving aroma formation in rice. However, interactions of both these elements with the complex process of 2-AP formation remain to be explored.

## Introduction

Aromatic rice is world famous due to its pleasant smell and enchanting flavor. Its “popcorn-like” smell is desired by consumers, so aromatic rice fetches premium prices in markets [1,2]. Among the fragrant rices, the Pakistani and Indian ‘Basmati’ rice and Thai ‘Jasmine’ rice are among the most popular and widely recognized [3].

Through instrumental analyses, researchers have identified approximately 200 different volatile compounds that contribute to rice aroma. For example, Buttery et al. [4] reported that 2-acetyl-l-pyrroline, (E)-2-nonenal, octanal, decanal, (E,E)-2,4-decadienal, nonanal, hexanal, 4-vinylphenol, and 4-vinyl-guaiacol are the key contributors to rice aroma while Jezussek et al. [5] argued that 4,5-epoxy-(E)-2-decenal, and 2-amino acetophenone are also important rice aroma compounds, which were previously unknown. The aroma profiles of various fragrant rice cultivars may be the same, but their levels may significantly vary [6]. The contributions of 13 volatile compounds suggested by Yang et al. [7] may affect aroma variability within different rice cultivars; this effect is also important.

Among these complicated multiple volatile substances that have been detected in fragrant rice, 2-acetyl-1-proline (2-AP) was identified as the most prominent compound contributing to aromatic characters of scented rice [8–10]. It is found in all plant parts of aromatic rice and some non-aromatic rice cultivars, except the roots [11]. However, in non-aromatic rice, 2-AP concentrations are substantially lower than in aromatic rice [12]. Although 2-AP creates aroma in several types of food, its role in rice is highly valued [13].

The factors affecting 2-AP biosynthesis are complex, and there are intricate pathways of genes whose expression responds to environmental factors and crop management practices [14–16]. However, controversies still exist regarding the effects of gene expression on aroma formation. For example, evidence from previous studies showed that nitrogen fertilizer affects aroma formation in aromatic rice and showed a significant relationship with 2-AP biosynthesis [17,18]. However, Itani et al. [19] reported that the application of nitrogen did not affect grain 2-AP concentrations in aromatic rice. In contrast, Suwanarit et al. [20] found that the concentrations of nitrogen in grain were negatively correlated with the aroma in the fragrant rice cultivar ‘KhawDauk Mali-105’. Similarly, a significant negative correlation was observed between 2-AP concentrations and hours of sunlight [21], which was later confirmed by Mo et al. [10], who declared that shade during the grain filling period led to a significant increase in 2-AP in rice grains. Additionally, the effects of temperature, irrigation regimes, drought stress, and salinity on the accumulation and biosynthesis of 2-AP in rice are widely reported [18, 22–24]. Furthermore, variation in 2-AP concentrations due to plant growth regulators, planting density, harvesting time and storage conditions has also been reported [25–27].

Both Zinc (Zn) and Lanthanum (La) are important elements affecting aroma formation in scented rice [17,28,29]. Application of both these elements through the soil (as base fertilizer) or foliar application (as an exogenous spray) improved 2-AP concentrations in aromatic rice [30–33]. However, the majority of these studies were conducted in an open environment where multiple factors may affect aroma formation. Therefore, an *in vitro* study is important to exclude interfering external factors and to gain insight regarding the roles of Zn and La in 2-AP biosynthesis and accumulation in grains. However, some earlier studies have investigated the effects of sucrose, glutamine and plant hormones on starch accumulation and on grain development of detached rice panicles *in vitro* [34,35,36], but no study has yet been reported regarding effects of 2-AP, Zn and La on 2-AP concentrations in detached rice panicles *in vitro*. Here, we conducted *in vitro* experiments to investigate the effects of 2-AP, Zn and La on the production and accumulation of 2-AP concentrations in detached aromatic rice panicles *in vitro*, while the accumulation and roles of proline, proline dehydrogenase (ProDH) was also assessed with respect to 2-AP formation.

## Materials and Methods

### Plant material and growing conditions

The seeds of three aromatic rice cultivars, Guixiangzhan, Pin14, and Pin15, were collected from the College of Agriculture, South China Agricultural University, Guangzhou, China. These cultivars are newly developed and locally famous due to their aroma. Before sowing, the seeds were soaked in water for 24 h and allowed to germinate in dark chamber at 30°C for next 24 h. All cultivars were raised at the Research Farm of the College of Agriculture, South China Agricultural University, Guangzhou (23°09′N, 113°22′E and 11 m above the sea level). 30-day-old seedlings were manually transplanted to the field at the recommended planting distance (20 cm × 20 cm). The soil of the experimental site was sandy loam containing soil organic matter 18.65 g kg^-1^, total nitrogen 1.17 g kg^-1^, available phosphorus 32.69 mg kg^-1^, available potassium 185.28 mg kg^-1^, and pH 6.44. This region has a humid subtropical monsoonal type of climate characterized by warm winters and hot summers with yearly average temperature range lies between 21–29°C.

### Plant sampling and in *vitro* culture preparation

At the beginning of the flowering stage, healthy panicles with consistent growth and uniform appearance were tagged and later collected two days after neck-panicle outgrowth from the flag leaf sheath, as described by Pan et al. [36]. The panicles, along with 20–22 cm of the main stem, were separated from rest of the plant and surface sterilized with sodium hypochlorite solution (1% *v/v*), washed with double distilled water and washed with deionized water. After washing, panicles, along with 12 cm of the main stem, were cut from the rest of the stem and cultured *in vitro*.

The basic *in vitro* culture medium was prepared as follows: 0.20 mmol L^-1^ CaSO_4_, 1.66 mmol L^-1^ K_2_SO_4_, 2.50 mmol L^-1^ KH_2_PO_4_, 0.30 mmol L^-1^ MgSO_4_, 20 mol L^-1^ FeC_6_H_5_O_7_, 5 mol L^-1^ H_3_BO_3_, 0.10 mol L^-1^ MnSO_4_, 0.005 mol L^-1^ (NH_4_)_6_Mo_7_O_24_, 0.05 mol L^-1^ CuSO_4_, 10 mmol L^-1^ NH_4_NO_3_, 160 mmol L^-1^ sucrose [34], and stored in wild mouthed culture bottles. The pH of the solution was maintained at 5.5.

### Preparation of 2-AP solution

The 2AP was synthesized by using the methods devised by Srinivas and Gurudutt [37]. The mother solution (1 mg of 2AP /ml in ethanol) with 92% purity was used in the culture medium.

### Treatments and sampling

The main experiment was divided into three sets: (i) the basic culture medium was supplied with two levels of 2-AP (0.503 ml L^-1^ and 1.028 ml L^-1^) and a control medium was prepared (without 2-AP). These media were represented as AP1, AP2 and AP0, respectively. (ii) The basic culture medium was supplied with three different levels of Zn by adding ZnCl_2_ (0.068 mg L^-1^, 14 mg L^-1^ and 30.10 mg L^-1^), and a control was prepared (without Zn). These were called Zn1, Zn2, Zn3, and Zn0, respectively. (iii) La was added to the basic culture medium at 0.015 mg L^-1^ and 0.270 mg L^-1^ (La1 and La2), and a control without La was prepared (La0). The treatment concentrations were based on pre-experimental studies form our lab Huang et al. [31] and Xiao et al. [38] in which several lower and higher levels of ZnCl_2_ and LaCl_3_ were used i.e., 0, 20, 40, 60, 80, 100, 120, 140, 160, 180 and 200 mg/kg of soil as Zn and La source in two different experiments with similar levels of Zn and La. It was found that 2-AP contents in grains were increased linearly from lower to medium levels of both Zn and La while decreased drastically at higher levels. The lowest values for 2-AP in rice grains were recorded at highest levels of Zn and La. The bottles were placed in a growth chamber (PGX-600A-3HR, Zhejiang, China) under standard conditions (16 h photo-period at 150–200 μmol m^-2^ s^-1^ light intensity) 25°C day/night temperature and 70% RH. The culture solution was renewed after every 2 d. Grains were sampled for biochemical analyses at 7 and 14 days after culture (DAC).

### Observations

The 2-AP concentrations in grains were tested according to the method of Tang and Wu [30] by using Thermo Finnigan TRACE 2000. The concentrations were calculated as described by Xiao et al. [38]. Briefly, the harvested grains were air dried, weighed 2 g and put into a 100 ml narrow-mouth(ed) bottle and added 15 ml of 4% KOH then sealed with plastic film, coated with wax seal, and kept for 2 days. Then, the gas sample was injected into the Thermo Finnigan TRACE-2000 Gas Chromatograph (GC) equipped with a DM-FFAP column (30 m × 0.32 mm × 0.25 μm) for 2-AP analysis. The temperature of the GC oven was 50°C (run for 3 min), which increased by 5°C/min to 120°C and was held at 65°C for 5 min, then increased to 200°C by 10°C/min and held at 200°C for 5 min. The flame ionization detector was adjusted to 230°C, with 30 ml/min flow rate of H_2_, for 350 ml/min air, 30 ml/min tail gas velocity. The retention time for 2AP was 35.62 min.

The grain proline concentrations were measured according the method of Bates et al. [39]. Briefly, grains (0.2 g) were homogenized in 5 ml of 3% sulfosalicylic acid. The crude extract was then heated at boiling water bath for 10 min. After that, the crude extract was placed in tap water to cool it and centrifuged at 5000 rpm for 10 min at 4°C. Two ml of the supernatant was mixed with 3 ml of 2.5% ninhydrin reagent and 2 ml of glacial acetic acid. The mixture was heated in a boiling water bath for 30 min and placed in a cool water bath to cool it before being extracted with 5 ml of toluene. The toluene extraction was then allowed to settle down for solution stratification. The absorbance of the red chromophore in the toluene fraction was measured at 520 nm. The amount of proline was determined by comparison with a standard curve and was expressed as micrograms per gram fresh weight (μg g^-1^ FW).

Proline dehydrogenase (ProDH) activity in grains was measured as described by Tateishi et al. [40] and Ncube et al. [41] with some modifications. Briefly, grains (0.2 g) were homogenized in 2 ml of 0.1 mol L^-1^ phosphate buffer (pH 7.4) containing 0.5% (v/v) TritonX-100. The homogenate was then centrifuged at 3000 rpm for 10 min at 4°C. The supernatant was used for enzyme assays. 0.2 ml of the supernatant was added to 0.3 ml of reaction mixture containing 15 mmol L^-1^ L-proline, 0.01 mol L^-1^ cytochrome c, 0.1 mol L^-1^ phosphate buffer (pH 8.0), 0.5% (v/v) TritonX-100. The reaction mixture was incubated at 37°C for 30 min, and the reaction was terminated by adding 0.5 ml of 10% trichloroacetic acid (TCA). Then, 0.3 ml of 0.5% 2-aminobenzaldehyde in 95% ethanol was added. The mixture was further incubated at 37°C for 10 min, centrifuged at 8000 rpm for 10 min, and absorbance of the colored supernatant complex was read at 440 nm.

To determine the Zn and La concentrations in grains, samples (0.1 g) were digested in a 5 ml mixture of HNO_3_:HClO_4_ (4:1 v/v). The digested solution was then used to measure the Zn concentration by using the method of Fang et al. [42]. The concentration was expressed as milligrams per gram dry weight (mg g^-1^ DW). Moreover, the La concentrations in grains was measured by using the inductively coupled plasma–mass spectrometry ICP-MS (JIS K 0133–2000). Grain La concentrations were too low to be measured, so data regarding grain La concentrations have not been presented.

### Experimental design and statistical analyses

Treatments were arranged in completely randomized design (CRD) in triplicate for each set of experiments, and there were twenty panicles in each treatment. Data (for each set of experiments) were analyzed with one-way analysis of variance (ANOVA) by using SPSS version 16.0 (SPSS, Inc., Chicago, IL, USA). The differences between treatments were separated by Duncan’s multiple range (DMR) test at a 5% probability level (P ≤ 0.05).

## Results

### Effect of 2-AP supplementation on 2-AP concentration in grains of detached aromatic rice panicles *in vitro*

**Fig 1:**
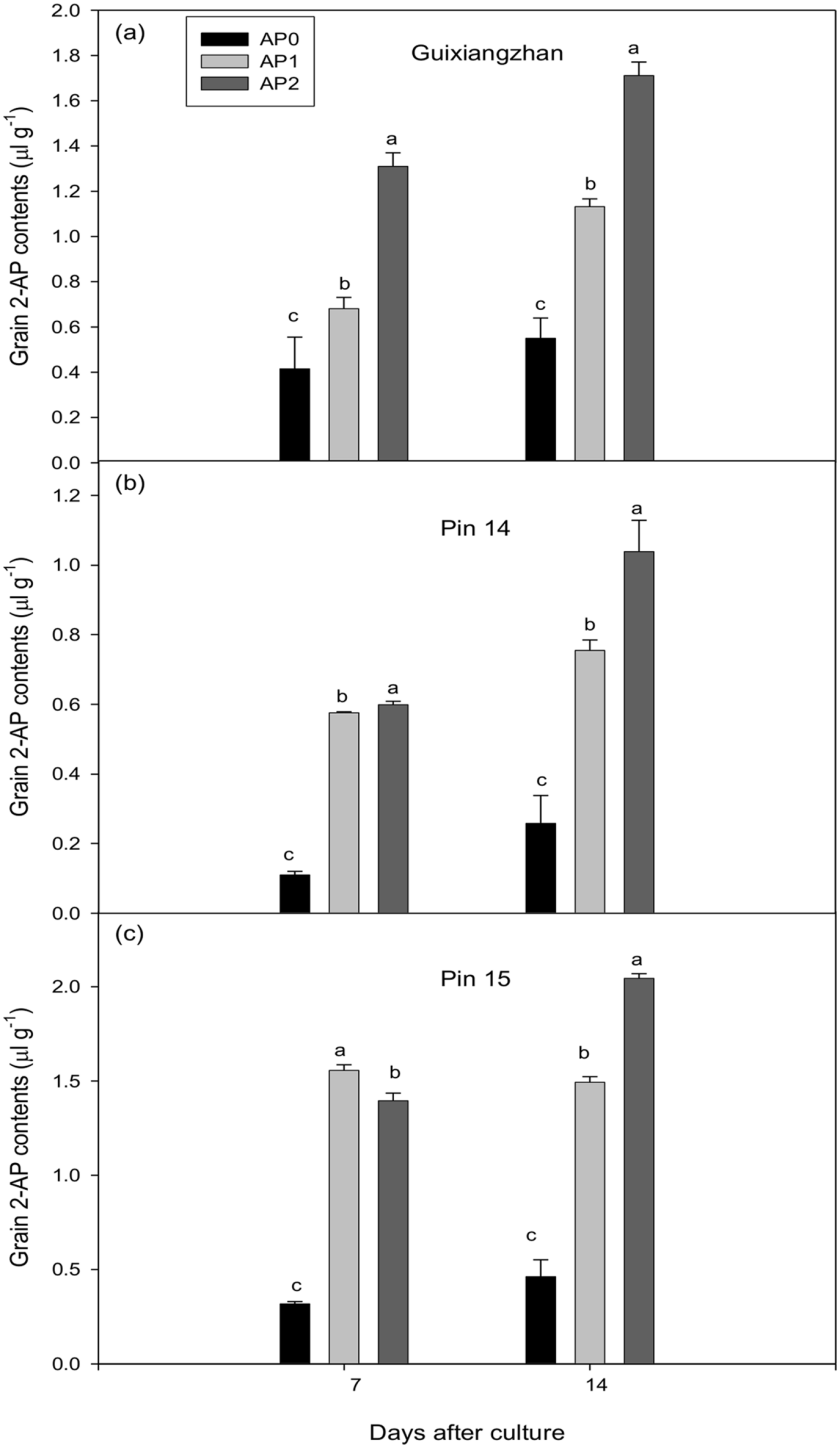
Effect of 2-AP supplementation on accumulation of 2-AP concentrations (μl g^-1^) in grains of detached aromatic rice panicles *in vitro*. Means in the same harvested stage by different lower case letters for the same variety differ significantly at P ≤ 0.05 by DMR test. Capped bars above means represent S.E. of three replicates.

The 2-AP supplementation in the basic culture medium had significant effects on the accumulation of 2-AP in grains. Increasing 2-AP supplementation was associated with increased 2-AP concentrations in grains for Guixiangzhan and Pin 14 at both 7 and 14 DAC and for Pin 15 at 14 DAC. For Pin 15 highest grain 2-AP concentrations were recorded at AP1 at 7 DAC followed by AP2 and AP0. Overall, the 2-AP concentrations in grains of detached aromatic rice panicles *in vitro* were increased significantly by 2-AP supplementation which resulted in 64.10–215.66%, 192.64–444.55% and 223.16–389.31% more 2-AP concentrations than AP0 (control) in Guixiangzhan, Pin 14 and Pin 15, respectively (Fig 1).

### Effect of Zn on 2-AP, Zn, proline concentration, and ProDH activity in grains of detached aromatic rice panicles in *vitro*

**Fig 2:**
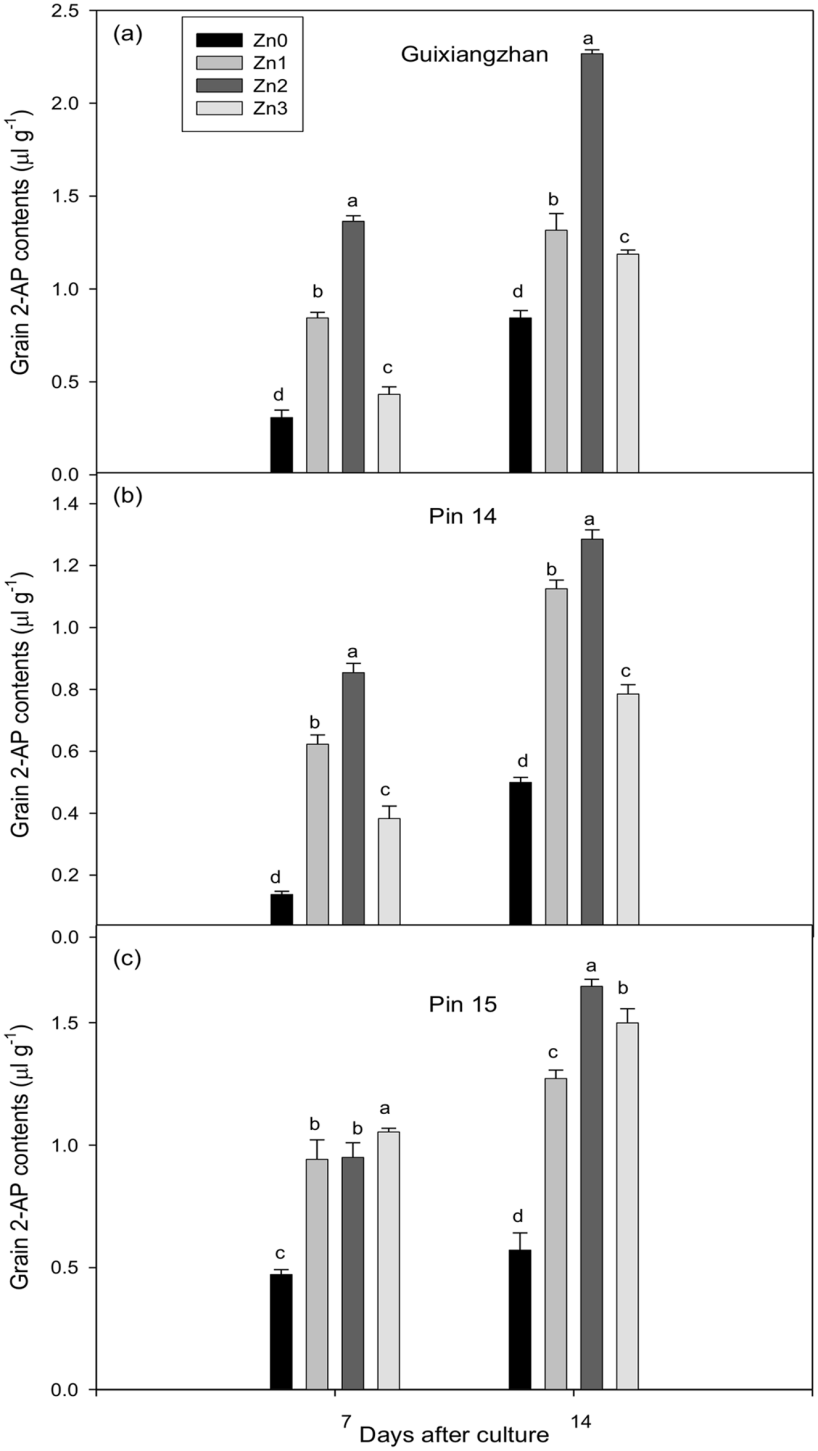
Effect of Zn supplementation on accumulation of 2-AP concentrations (μl g^-1^) in grains of detached aromatic rice panicles *in vitro*. Means in the same harvested stage by different lower case letters for the same variety differ significantly at P ≤ 0.05 by DMR test. Capped bars above means represent S.E. of three replicates.

Significant increase in 2-AP concentrations in rice grains supplemented with Zn were measured for all fragrant rice cultivars analyzed at both 7 and 14 DAC relative to Zn0 (control). Compared to Zn0 (control), the 2-AP concentrations in grains increased by 40.58%-342.86%, 57%-518.84% and 100%-188.97% for Guixiangzhan, Pin 14 and Pin 15, respectively. The trends for Guixiangzhan and Pin 14 were recorded as Zn2>Zn1>Zn3>Zn0 at both 7 and 14 DAC. For Pin 15 at 7 DAC, increasing Zn supplementation was associated with increased 2-AP concentration in grains of detached aromatic rice panicles *in vitro* (Fig 2).

All Zn treatments increased the Zn concentrations in grains of all varieties at both 7 and 14 DAC significantly. The trend for Zn accumulation among varieties was observed as: Pin 14 > Pin 15 > Guixiangzhan. Furthermore, for Guixianzhan and Pin 15, highest grain Zn concentrations were recorded at Zn3 and Zn1 at both 7 and 14 DAC, respectively, while for Pin 14, highest grain Zn concentrations were recorded at Zn2 and Zn3 at both 7 and 14 DAC, respectively. Further, the proline concentrations in grains for Pin15 at both 7 and 14 DAC and for Pin14 at 7 DAC were dramatically increased by all the Zn treatments. In Guixiangzhan, significant improvements in proline concentration were observed for Zn1 and Zn3 at 7 DAC and for Zn1 and Zn2 at 14 DAC. In addition, for Pin 14, a significant increase in proline concentrations was observed for Zn1 and Zn3, but not in Zn2 at 14 DAC. Additionally, the ProDH activity in grains for all cultivars varied for different Zn treatments. The ProDH activities in grains for Guixiangzhan, Pin14 and Pin 15 at 14 DAC were found to be significantly increased by Zn supplementation. Moreover, Zn1 and Zn2 significantly increased ProDH activities in grains for Guixianzhan and Pin 14 at 7 DAC were also recorded. Additionally, Zn1 significantly increased ProDH activity in grains for Pin 15 at 7 DAC (Table 1).

**Table 1:** Effect of Zn supplementation on Zn and proline contents as well as ProDH activities in grains of detached aromatic rice panicles *in vitro*.

Cultivar	Treatments	Zn contents (mg g^-1^ DW)		Proline contents (μg g^-1^ FW)		ProDH activity (U g^-1^ FW)	
		7 DAC	14 DAC	7 DAC	14 DAC	7 DAC	14 DAC
Guixiangzhan	Zn0	0.097 c	0.093 d	18.94 c	19.24 b	0.554 b	0.185 c
	Zn1	0.108 b	0.118 c	21.06 b	25.92 a	0.627 a	0.461 b
	Zn2	0.110 b	0.128 b	18.33 c	26.61 a	0.646 a	0.480 b
	Zn3	0.119 a	0.135 a	22.58 a	20.76 b	0.609 ab	0.627 a
Pin 14	Zn0	0.143 b	0.145 c	12.77 c	23.49 c	0.812 b	0.166 d
	Zn1	0.145 b	0.149 bc	25.72 a	26.73 a	0.923 a	0.258 c
	Zn2	0.166 a	0.164 b	24.30 a	23.19 c	0.978 a	0.424 a
	Zn3	0.159 ab	0.183 a	15.60 b	25.11 b	0.812 b	0.369 b
Pin 15	Zn0	0.096 c	0.090 c	16.10 c	21.20 c	0.517 b	0.166 c
	Zn1	0.144 a	0.141 a	22.78 a	24.91 b	1.070 a	0.369 a
	Zn2	0.130 b	0.125 b	22.23 a	29.26 a	0.554 b	0.351 a
	Zn3	0.124 b	0.129 b	19.75 b	30.37 a	0.535 b	0.314 b

Means in the same column followed by different lower case letters for the same variety differ significantly at P ≤ 0.05 by DMR test. DAC = days after culture. FW = fresh weight; DW = dry weight

### Effect of La on 2-AP concentration, and ProDH activity in grains of detached aromatic rice panicles *in vitro*

**Fig 3:**
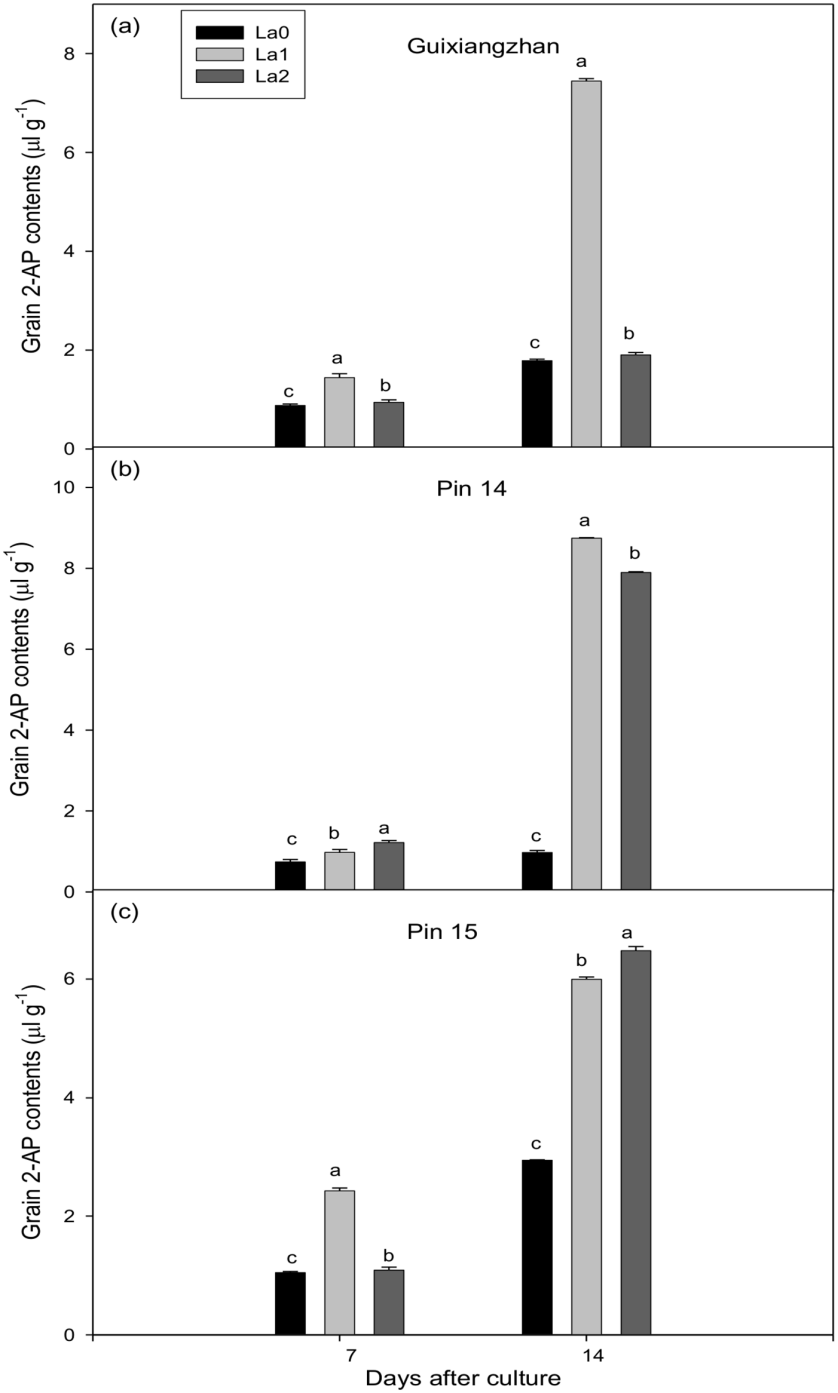
Effect of La supplementation on accumulation of 2-AP concentrations (μl g^-1^) in grains of detached aromatic rice panicles *in vitro*. Means in the same harvest stage with different lower case letters for the same variety differ significantly (P ≤ 0.05) according to the DMR test. Capped bars above the means represent the S.E. of three replicates.

The results showed that all La treatments significantly increased the grain 2-AP concentrations for Guixiangzhan, Pin 14 and Pin 15 at both 7 and 14 DAC, with increases of 6.49–316.51%, 32.03–799.595%, and 4.10–131.27% for Guixiangzhan, Pin 14, and Pin 15 compared to La0 (control), respectively. Increasing La supplementation was associated with increased 2-AP concentrations in grains for all varieties under study. However, the highest La level resulted in reduced 2-AP concentrations in rice grains. The overall trend for La-induced increment in 2-AP concentrations in rice grains was La1> La2 > La0 (Fig 3).

The La treatments increased ProDH activity in grains, but not at La2 for Pin 14. A significant increase in ProDH activity in grains for Pin 15 at both 7 and 14 DAC was found at all La levels. For Pin 14, a significant increase in ProDH activity in grains was observed for La1 at both 7 and 14 DAC. Furthermore, substantial increase in ProDH activity in grains was also identified at La1 at 7 DAC for Guixiangzhan (Table 2).

**Table 2:** Effect of La supplementation on ProDH activity (U g^-1^ FW) in grains of detached aromatic rice panicles *in vitro*.

Gultivar	Treatment	7 DAC	14 DAC
Guixiangzhan	La0	0.387 b	0.277 a
	La1	0.701 a	0.314 a
	La2	0.535 b	0.369 a
Pin 14	La0	0.351 b	0.332 b
	La1	1.181 a	0.443 a
	La2	0.295 b	0.221 c
Pin 15	La0	0.498 c	0.240 c
	La1	1.199 a	0.535 a
	La2	0.867 b	0.387 b

Means in the same column followed by different lower case letters for the same variety differ significantly at P ≤ 0.05 by DMR test. DAC = days after culture.

### Correlation analysis among investigated parameters

Significant correlations were observed between 2-AP concentration in grains at 7 and 14 DAC in response to 2-AP supplementation. Furthermore, under Zn supplementation, 2-AP in grains at 7 DAC is significantly correlated with 2-AP at 14 DAC whereas negative but non-significant associations of Zn concentrations with grain 2-AP were observed at 7 and 14 DAC. Moreover, grain Zn concentrations at 7 DAC were significantly and positively correlated with grain Zn concentrations at 14 DAC. Likewise, grain proline concentrations were also significantly associated with grain 2-AP concentrations at both 7 and 14 DAC whereas ProDH was significant at only 14 DAC. Additionally, significant correlation between ProDH activity at 7 and 14 DAC was measured under La supplementation (Table 3).

**Table 3:** Correlation analyses among different indices in three sets of experiments at 7 and 14 DAC of rice panicles *in vitro*.

Experimental sets		Index		2-AP		Zn		Proline		ProDH	
				7 DAC	14 DAC	7 DAC	14 DAC	7 DAC	14 DAC	7 DAC	14 DAC
Set (i)	2-AP supplementation	2-AP	7 DAC	1	0.9350**	-	-	-	-	-	-
			14 DAC	0.9350**	1	-	-	-	-	-	-
Set (ii)	Zn supplementation	2-AP	7 DAC	1	0.9208**	-0.0399	-0.0096	0.4253	0.6611*	-0.0005	0.4363
			14 DAC	0.9208**	1	-0.0853	0.0161	0.4388	0.5828*	-0.0987	0.5907*
		Zn	7 DAC	-0.0399	-0.0853	1	0.9270**	0.2119	0.2548	0.7998*	0.0969
			14 DAC	-0.0096	0.0161	0.9270**	1	0.1172	0.2736	0.6940*	0.3086
		Proline	7 DAC	0.4253	0.4388	0.2119	0.1172	1	0.2029	0.3154	0.4399
			14 DAC	0.6611*	0.5828*	0.2548	0.2736	0.2029	1	0.0023	0.1109
		ProDH	7 DAC	-0.0005	-0.0987	0.7998*	0.6940*	0.3154	0.0023	1	0.042
			14 DAC	0.4363	0.5907*	0.0969	0.3086	0.4399	0.1109	0.042	1
Set (iii)	La supplementation	2-AP	7 DAC	1	0.4025	-	-	-	-	0.5846	0.5808
			14 DAC	0.4025	1	-	-	-	-	0.5667	0.2485
		ProDH	7 DAC	0.5846	0.5667	-	-	-	-	1	0.8787*
			14 DAC	0.5808	0.2485	-	-	-	-	0.8787*	1

* Significant at P ≤ 0.05

** Significant at P ≤ 0.01

DAC = days after culture

## Discussion

2-AP biosynthesis and accumulation in rice is an important phenomenon that is affected by several factors. Culturing the detached rice panicles *in vitro* is an efficient way to study the main factors affecting the aroma without being influenced by sub-factors. Previously, Liang et al. [34] and Pan et al. [36] have reported the effects of sucrose, glutamine and plant hormones on starch accumulation and on the grain development of detached rice panicles *in vitro*. As expected, we have successfully investigated the effects of 2-AP, Zn and La on 2-AP concentration of detached aromatic rice panicles *in vitro* as well as the enzymes involved in the pathways leading to 2-AP biosynthesis.

**Fig 4:**
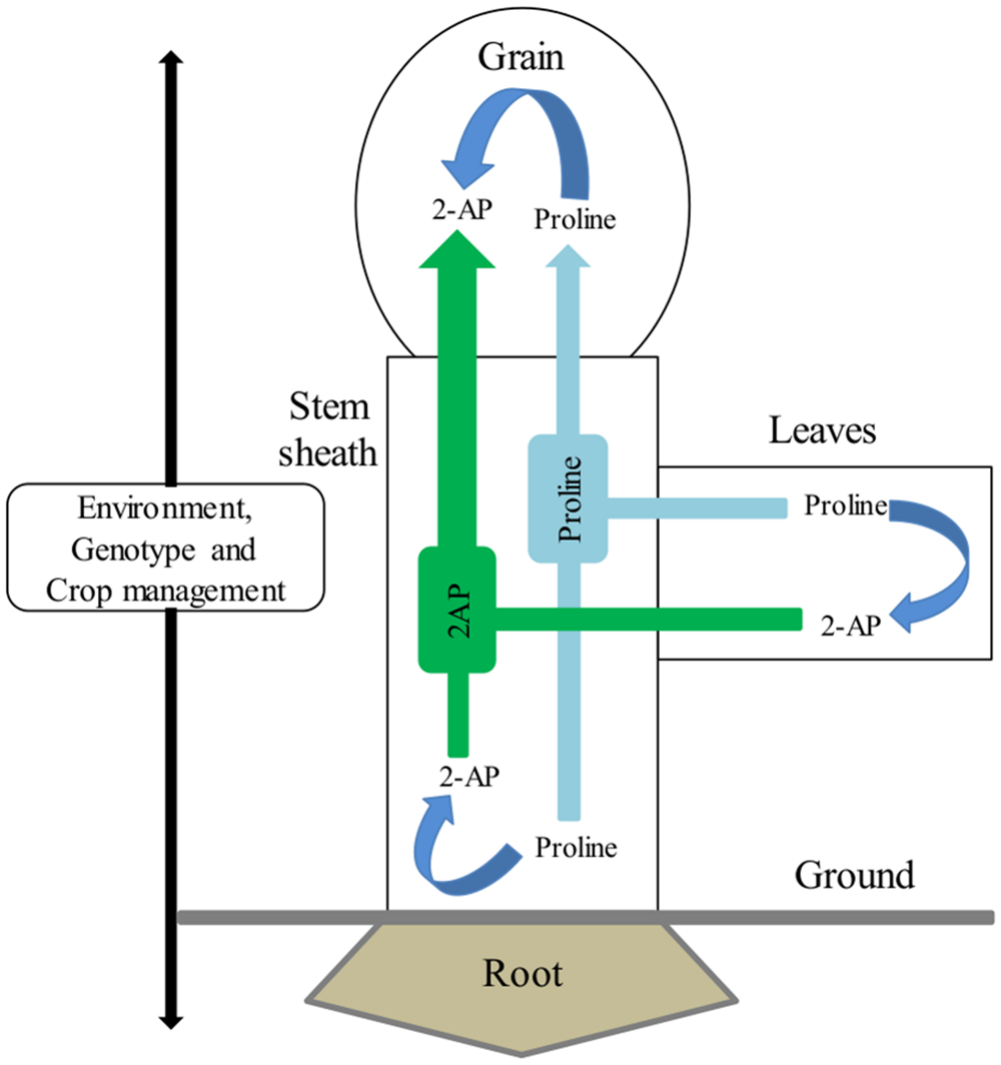
A theoretical illustration of 2-AP formation and transportation in aromatic rice plants. Proline might be converted to 2-AP in the stem sheet and in leaves and grains before accumulating in grains, or proline might accumulate directly in the grains, where it is further converted into 2-AP through various biochemical pathways. Environmental factors, crop genotype, and crop management practices affect rice aroma.

In this study, we have found that the supplementation of 2-AP in culture medium significantly increased grain 2-AP concentrations (Fig 1). This dramatic increase of 2-AP in grains might possibly due to direct transportation of 2-AP from culture medium (supplemented with 2-AP) towards rice panicles. Translocation of 2-AP from above ground plant parts towards grains contribute significantly in grain 2-AP concentrations [11,43]. Our findings also suggest that increased 2-AP concentrations in detached panicles might be due to direct translocation from the culture medium to panicles and indirect translocation from other parts of the plant such as leaves, stems or sheaths. Proline might be converted into 2-AP in stem sheaths and leaves before being carried to grains, where the same mechanism is already occurring. A pictorial view of this mechanism is presented in Fig 4. Thus, the accumulation of 2-AP in grains might be attributed to the transportation of 2-AP from the leaves and stem sheaths to the grains of the aromatic rice plant.

Zn and La are two important elements that are applied to rice through fertilizer as micro-nutrients; however, their contributions to 2-AP in rice grains are also significant. Our previous experiments conducted in an open environment [30,32,33,38] show that the application of Zn and La significantly contributes to grain 2-AP. Here, we also observed that Zn treatments led to a considerable increase in the production of 2-AP in grains (Fig 2), but moderate La levels were also found for grain 2-AP *in vitro* (Fig 3). Furthermore, not only 2-AP, Zn treatments also increased the Zn concentration in grains at both 7 and 14 days after cultured for the three varieties (Table 1). Proline is a precursor of 2-AP, which makes it important. Tang and Wu [30] also reported that application of Zn and La increased proline concentrations in leaves. Likewise, higher 2-AP concentrations in rice leaves at the vegetative stage are correlated with leaf proline concentrations rather than γ-aminobutyric acid (*GABA*) [22]. Our results also suggested that Zn and La significantly increased the proline concentrations in grains of detached panicles, and that proline might be transported from other parts towards grains. Transportation of proline from other parts towards grains as well as already converted 2-AP concentrations in grains is equally important to increase grain 2-AP concentrations. A diagram of the transportation of proline and 2-AP is shown in Fig 4.

Significant increases in grain 2-AP concentrations at all levels of Zn treatment relative to control were observed for all fragrant rice cultivars analyzed, both at 7 and 14 DAC (Fig 2). Higher ProDH activity in grains was also detected for some Zn (Table 1) and La treatments (Table 2). The significant relationships were also observed of 2-AP, Zn and La with grain 2-AP concentrations (Table 3). Zn and La affected the ProDH activity resulted in proline conversion to Δ^1^-pyrroline-5-carboxylic acid then converted to 2-AP [44]. Our observations regarding Zn and La induced changes in grain 2-AP concentrations of detached aromatic rice panicles *in vitro* tell us that both elements may be involved in complicated biosynthetic mechanisms of aroma formation directly, or the elements might have indirect effects on 2-AP biosynthesis through regulation of enzymatic activities involved in 2-AP formation. Furthermore, the biochemical pathways leading to 2-AP biosynthesis are quite complicated and have not been fully resolved yet. Different mechanisms have been reported. For instance, the pathway might work as follows: conversion of proline, glutamic acid and ornithine to Δ^1^-pyrroline-5-carboxylic acid by proline dehydrogenase, pyrroline-5-carboxylic acid synthetase and ornithine aminotransferase, respectively, followed by conversion to 2-AP *via* enzymatic (acetyl-CoA groups) or non-enzymatic (methylglyoxal) pathways [31]. Secondly, ornithine decarboxylase may convert ornithine to putrescine, diamine oxidase may oxidize putrescine to form γ-aminobutyl aldehyde (*GABald*) and γ- amino butyric acid (*GABA*) by inactivation of betaine aldehyde dehydrogenase (*BADH2*) to form Δ^1^-pyrroline, which then converts to 2-AP with the same enzymatic and non-enzymatic pathways described previously [45–48]. Proline is regarded as the precursor of 2-AP formation in rice, but *GABald* is possibly the direct precursor of 2-AP biosynthesis via *BADH2* [46].

Additionally, micronutrients such as Zn and La might have some physiological functions which may be catalytically active cofactors in enzymes, or may have enzyme-activating functions that play important roles in 2-AP biosynthesis [49,50]. However, considering the intricate pathways of 2-AP biosynthesis, there are still several enzymes yet to be characterized [9,51]. For example, it remains to be determined how Δ^1^-pyrroline-5-carboxylic acid is converted to Δ^1^-pyrroline and how Δ^1^-pyrroline together with acetyl-CoA groups in an enzymatic way to form 2-AP.

Overall, our results indicate that higher grain 2-AP concentrations in detached rice panicles cultured *in vitro* seem to be due to increased transportation of 2-AP from the culture medium into grains. The increased proline concentrations and ProDH activities are due to Zn and La supplementation. Application of both Zn and La at low levels might be helpful for enhancing rice aroma, and this supplementation can be easily practiced. To explore the possible roles of Zn and La in rice aroma formation and its interactions with physico-chemical processes of 2-AP biosynthesis, intensive research is needed at the molecular level.

## Conclusions

In this study, we have demonstrated that the application of 2-AP, Zn and La significantly increases 2-AP concentrations in detached rice panicles *in vitro*. Increased 2-AP concentrations might be attributed to higher rates of proline formation and increased ProDH activities. Transportation of 2-AP directly from the culture medium and increased proline concentrations due to Zn and La supplementation might also be responsible for the higher grain 2-AP concentrations. Thus, Zn and La might have some relationship with 2-AP formation, but studies are still needed at the molecular level to understand exactly how these nutrients are involved in 2-AP formation.
